# Influence of gender and oral health knowledge on DMFT index: a cross sectional study among school children in Kaski District, Nepal

**DOI:** 10.1186/s12903-023-02755-z

**Published:** 2023-02-01

**Authors:** Kamal Prasad Chapain, Krishna Gopal Rampal, Kalpana Gaulee Pokhrel, Chiranjivi Adhikari, Deependra Hamal, Khem Narayan Pokhrel

**Affiliations:** 1 Development and Research Service International Nepal, Khumaltar, Lalitpur, Nepal; 2 Department of Medical Science, The University of Cyberjaya, Cyberjaya, Malaysia; 3 School of Health and Allied Sciences (SHAS), Pokhara University, Kaski, Nepal; 4 Department of Public Health, Indian Institute of Public Health-Gandhinagar (IIPHG), Gandhinagar, Gujarat India; 5 Manipal College of Medical Sciences, Pokhara, Nepal

**Keywords:** Dental caries, Oral health knowledge score, Oral health, School children, Nepal

## Abstract

**Background:**

Oral health problems are highly prevalent among school children in Nepal. Poor oral health condition may be influenced by various factors. However, little is known about the sociodemographic and awareness related factors on oral health problems among school children in Nepal. Therefore, this study aimed to assess the association of gender and knowledge on DMFT index among school children.

**Methods:**

A cross-sectional study was conducted among school children of Grade Seven in 12 schools of Kaski district in Nepal. Schools were randomly selected from the urban and semi-urban areas in the district. Data were collected covering oral health knowledge, socio-demographic characteristics, oral health condition and practices. The factors of poor oral health condition and practices were examined using t-test, one-way ANOVA, and multiple linear regression.

**Results:**

Of the total participants (n = 669), 54.9% were females and their mean DMFT score was 1.82 (SD = 1.07). Total decayed score was higher among those who did not have knowledge that fluoride prevents decay compared to those who had knowledge about it (Being aware of fluoride prevents decay: Mean = 1.21 (SD = 1.54) versus not being aware of that: mean = 2.13 (SD = 2.13); p = 0.029). Females were more likely to have higher DMFT scores compared to males (β-coefficient = 0.43, 95% CI 0.13, 0.73, p = 0.005). In addition, higher knowledge score was negatively associated with higher DMFT score (β-coefficient = − 0.09, 95% CI − 0.20, -0.01, p = 0.047).

**Conclusion:**

Being female students and those having lower level of knowledge on oral health attributed to higher DMFT index. Periodic dental check-up coupled with oral health education on regular brushing, use of fluoridated paste, tongue cleaning and care of gum diseases are recommended in schools.

## Background

Oral health problems are common among school children. According to the World Health Organization (WHO), an estimated 60–90% of the school children suffer from dental caries worldwide [1]. Dental caries is one of the most prevalent conditions, ranking the first for the decay of permanent teeth (2.3 billion people) and 12th for deciduous teeth (560 million children) [2]. Moreover, children suffering from oral diseases are 12 times more likely to be restricted in daily activities than the normal children having no oral diseases [3].

Oral health problems are usually multifactorial. Socioeconomic factors are attributable to the severity and wider distribution of oral diseases [4]. In addition, family environment, including socio-behavioral and environmental factors are strongly related with the oral health condition of the children. Clearly, it has been shown that children's poor oral health condition is related to lower level of parental education and the family income [4]. Children of parents who have lower educational level and lower level of socio-economic status are much more affected by dental caries and other oral health problems [3].

A supportive environment should be provided for the promotion of oral health education in the community as well as in the schools and policy makers should plan in such a way that social discrimination can be reduced in school settings [5]. Healthy oral heath behavior minimizes the risk of various oral health problems. Brushing twice a day with fluoride toothpaste is one of the most important habits for good oral health. Children can learn about the benefits of good oral hygiene through brushing during the day and night after meal. Children are also needed to be taught to brush their teeth twice daily with fluoride toothpaste [6].

Healthy oral health practice is a key to prevent oral health problems. A study conducted in Nepal has shown that about 95% of the school children had practice of brushing their teeth at least once a day and 71% of those used fluoridated tooth-paste. It was also found that during the last 6 months, only 4% of the school children received oral health services [7]. To address low level of awareness and poor oral hygiene practices, school oral health promotion is essential. Although dental caries is a highly preventable disease, a high proportion of the people in Nepal who are poor and marginalized are suffering from it [8]. In addition, poor oral hygiene is regarded as one of the most important risk factors for common oral diseases. To add further, 58% of the schoolchildren aged between 5–6 years were affected by dental caries. The study also revealed that oral health problems were the prime reason of missing school in Nepal, where 32% of the students missed their school due to dental pain [9].

Evidence is available about the association of oral health problems with oral hygiene practices and sociodemographic factors. A study conducted in Nepal has shown that socio-economic factors and poor oral health practices resulted in oral health problems [10]. Another study has also shown that gingival bleeding was common in children in rural areas [11]. However, evidence is limited about the knowledge on oral health, gender and proper oral health practices and its association with oral health condition among school children. Therefore, this study aimed to assess the association of knowledge, practices and gender with DMFT index.

## Methods

### Study design and setting

This study is the part of the school-based oral health intervention study to provide oral health services and counseling to school children. The baseline of the intervention study was conducted among school children of Grade Seven in 12 community schools of Kaski district in Nepal. The district is the provincial capital of Gandaki Province, Nepal. We selected the schools in the urban and semi-urban areas of the district. We purposively selected Kaski district as the study district, where people with diverse population from more than 15 districts of its catchment areas reside. The selection of schools was done in semi-urban areas of the district considering the higher prevalence of oral health problems in the community schools.

### Sampling strategy and sample size

We randomly selected 12 government schools out of 325 schools. All of children in Grade Seven of the selected schools were included in the study. The study was a part of an intervention study about oral health counseling and promotion targeting basic level schools. The rationale for selecting students of Grade Seven was that the school children of higher grade at basic level schools would adopt the intervention well. We did not take the highest grade of basic level schools as the students of Grade Eight would graduate to secondary schools and our intervention would not be able to capture those. We took the reference prevalence of dental caries as 60% among school children while calculating the estimated sample size [9]. We also considered 95% confidence interval and 80% of the power of the study. The estimated sample size was 654 and we increased the sample size to 669 in this study considering various characteristics to be studied.

### Participant’s inclusion and exclusion criteria

The school children, who were enrolled in the selected schools at Grade Seven at least six months prior to the study were included. Those who were not able to respond the questions because of their intellectual disability were excluded from the study. Also, those who were absent at the time of data collection were excluded in this study.

### Study tools and measurements

#### Sociodemographic characteristics, oral health knowledge, and practices

Information on students’ sociodemographic characteristics, visiting dentist or dental care practitioners for their services, student’s knowledge on oral health, oral health practices and oral health seeking behavior were obtained using various set of questionnaires.

Student’s oral health knowledge was scored using the similar methods adopted from previous studies [12]. Oral health knowledge was determined by asking the following questions: (1) periodontal disease can affect health, (2) regular tooth brush can protect tooth decay, (3) fizzy soft drinks affect the teeth, (4) use of fluorides prevent tooth decay, (5) gingivitis is a disease that makes your gums bleed, (6) proper tooth brushing can prevent dental caries, (7) sugar causes tooth decay, (8) tooth decay is a disease that destroys your teeth, and (9) healthy teeth means strong and carries free teeth. The total oral health knowledge score was ranging from 0 to 9 being nine as the highest score. The response of the knowledge test was measured as: Yes = 1, No and Don’t Know = 0. For the reliability of items on the knowledge scale, we calculated the Cronbach’s alfa and the value was 78.6 in this study. For the validity of the items of questions, the English questionnaires were translated into Nepali and re-translated in English. The pre-testing was also done to ensure the validity of the questionnaires to assess how the participants would understand. Face validity was ensured after taking expert advice from dentists, researchers and school teachers in the schools. This knowledge scale has already been applied in India and Bangladesh, which are similar settings to the context of Nepali basic schools [12, 13].

Oral health practices were assessed by asking the following questions: (1) frequency of brushing teeth per day, (2) time spent for brushing in minutes, (3) cleansing aids used, (4) materials used to clean teeth, (5) frequency of changing tooth brush, (6) type of toothpaste used, (7) mouth rinsing after eating, and (8) cleaning tongue after meal or during brushing. The questionnaires were already applied in the context of Nepal [14].

*Oral Health Condition* (Decayed, Missing due to caries, and Filled Teeth in the permanent teeth (DMFT) Index).

Oral health problems of the school children were assessed using the Total DMFT Index Score and Total Decayed Score. The DMFT score numerically expresses the prevalence of dental caries and is obtained by calculating the number of Decayed (D), Missing (M) and Filled (F) teeth (T). It is used to get an estimate how much the dentition until the day of examination has become affected [15]. It is calculated for 28 (permanent) teeth, excluding third molar (wisdom teeth). Oral examination was carried out to determine: How many teeth had caries lesions (incipient caries not included), how many teeth were extracted and how many teeth had fillings or crowns. The total DMFT score was 28.

### Data collection

Oral health condition was examined by the dental surgeon, the first author himself. For data collection, two research assistants were assigned to collect data from schools between April and May 2021. Both research assistants were certified dental hygienist and had experience in dental health services for 5 years. They had experience of data collection in community and school settings as well. Prior to the data collection, the research assistants received three days training covering data collection and ethical procedures. Pre-testing of questionnaires was done in non-selected schools. Necessary modification in the questionnaires was made.

### Data analysis

We examined the association between factors and oral health condition, knowledge and practices of school children. We used t-test and one way ANOVA to describe the mean characteristics of decayed and DMFT scores. Multivariate analysis was conducted to determine the factors associated with DMFT scores after controlling for sociodemographic variables. We used SPSS version 25.0 for the entire data analysis process. We stored the collected data files in the locked cabinet of the first author, then the data were entered and stored in a password protected computer. For data analysis, we used SPSS 25.0 software.

***Ethical consideration***

Ethical approval was obtained from the Nepal Health Research Council (ERB Protocol Registration No. 165/2021PhD) and the University of Cyberjaya (UOC) Research Ethics Committee (CRERC Reference no UOC/CRERC/EXTERNAL/07/2020 -SPC620200605). Participants were ensured for their voluntary participation and confidentiality of information.

## Results

### Sociodemographic and oral health characteristics of the participants

We assessed the sociodemographic and oral health characteristics of 669 participants. Among them, 54.9% were females, the mean age of students was 13 (SD = 1) (not shown in table). About 67.1% had used oral health services. More than half (52.6%) had practices of brushing teeth two times a day and nearly half (49.3%) reported that their brushing time was 2–3 min. Among total, only 29.1% used fluoridated toothpaste. About 59.3% of the participants had decayed teeth and 3% had filled teeth. Their average knowledge score on oral health was 7.06 (SD = 1.46) and the mean DMFT score was 1.82 (SD = 1.07) (Table 1).

**Table 1:** Basic characteristics of participants (n = 669)

Characteristics	Total (n = 669)	%
*Gender*		
Male	302	45.1
Female	367	54.9
*Father's education*		
Illiterate	68	10.2
Literate and basic level	396	59.2
High school and above	205	30.6
*Mother's education*		
Illiterate	114	17.1
Literate and basic level	405	60.5
High school and above	150	22.4
*Father's occupation*		
Employed	303	45.3
Foreign employment	158	23.6
Agriculture	111	16.6
Business	81	12.1
Unemployed	16	2.4
*Mother's occupation*		
Employed	170	25.4
Foreign employment	23	3.4
Agriculture	220	32.9
Business	105	15.7
Unemployed	151	22.6
*Using health services or not*		
Yes	220	32.9
No	449	67.1
*Brushing aid*		
Datiwan/other	4	0.6
Brush	665	99.4
*Brushing material*		
Powder/coila/others	13	1.9
Toothpaste	656	98.1
*Brush change*		
Every 1 month	370	55.3
Every 3 month	156	23.3
Every 6 month	34	5.1
Bristle out of shape	109	16.3
*Brushing frequency*		
Two times a day	352	52.6
Once-daily	260	38.9
More than 2 times a day	20	3.0
Occasionally	37	5.5
*Brushing Time*		
Less than 1 min	155	23.2
2–3 min	330	49.3
More than 3 min	184	27.5
*Paste type*		
Fluoridated	195	29.1
Non fluoridated	30	4.5
Don’t know	444	66.4
*Mouth gargle after meal*		
Never	37	5.5
Sometimes	65	9.7
Always	567	84.8
*Tongue clean after meal*		
Never	132	19.7
Sometimes	400	59.8
Always	137	20.5
*Decay status*		
Non-decayed	272	40.7
Decayed	397	59.3
*Filled status*		
Filled	20	3.0
Not filled	649	97.0
Total knowledge score (mean, SD)	7.06	1.46
Total DMFT score (mean, SD)	1.82	1.07

### Total knowledge score, DMFT score and total decayed score by sociodemographic factors

We applied t-test to see the mean differences of DMFT score and Decayed score according to the sociodemographic characteristics. Total DMFT scores were significantly higher among females compared to those of males (Male: mean = 1.47 (SD = 1.78) versus female: mean = 1.90 (SD = 1.95); p = 0.003). Similarly, total decayed scores were also higher among females compared to males (Male: mean = 1.24 (SD = 1.53) versus female: mean = 1.62 (SD = 1.75); p = 0.003). Knowledge scores were also higher among females compared to males (Male: mean = 6.86 (SD = 1.62) versus female: mean = 7.25 (SD = 1.27); p < 0.001). Those who did not visit hospitals had lower DMFT scores compared to those who visited hospitals (Hospital visit: mean = 1.99 (SD = 1.99) versus No hospital visit: mean = 1.57 (SD = 1.82); p = 0.007). The knowledge level was lower among those who did not visit hospitals (Hospital visit: Mean = 7.25 (SD = 1.22) versus No Hospital Visit: mean = 6.97 (SD = 1.55); p = 0.018).

Knowledge level was higher among the children whose fathers had education level at high school and above compared to those whose parents were illiterate. (Father’s education high school and above: mean = 7.20 (SD = 1.30) versus illiterate: mean = 6.62 (SD = 1.95). (Table 2).

**Table 2:** Total Knowledge score, DMFT score and Decayed score, by sociodemographic factors

Characteristics	Total Knowledge score (Mean, SD)	p value	Total DMFT score (Mean, SD)	p value	Total Decayed score (Mean, SD)	p value
Gender		< 0.001		0.003		0.003
Male	6.86 (1.62)		1.47 (1.78)		1.24 (1.53)	
Female	7.25 (1.27)		1.90 (1.95)		1.62 (1.75)	
Father's education						
Illiterate and no schooling	6.62(1.95)	0.016	1.63 (1.88)	0.927	1.46(1.72)	
Literate and Basic	7.06 (1.42)		1.72 (1.87)		1.47(1.68)	0.866
High school and above	7.20 (1.30)		1.69(1.94)		1.40(1.64)	
Mother’s education						
Illiterate and no schooling	6.96(1.72)	0.601	1.87(2.13)	0.596	1.55(1.81)	
Literate and Basic	7.05(1.47)		1.68(1.86)		1.43(1.64)	0.760
High school and above	7.15(1.16)		1.65(1.77)		1.42(1.62)	
Father's occupation						
Employed	7.09(1.51)	0.960	1.71(1.85)	0.973	1.48(1.67)	
Foreign employed	7.06(1.40)		1.66(1.92)		1.42(1.75)	0.960
Business	7.07(1.42)		1.75(2.07)		1.49(1.68)	
Agriculture/other	7.05(1.39)		1.75(1.91)		1.38(1.62)	
Un-employed	6.56(1.54)		1.44(1.26)		1.25(1.06)	
Mother’s occupation						
Employed	7.06(1.42)	0.164	1.52(1.74)	0.635	1.32(1.63)	
Foreign employed	6.52(1.99)		1.87(2.16)		1.65(2.10)	0.753
Business	7.01(1.49)		1.81(2.11)		1.51(1.76)	
Agriculture/other	7.21(1.34)		1.72(1.81)		1.44(1.57)	
Un-employed	6.95(1.52)		1.80(1.97)		1.53(1.73)	
Dental checkup done						
Yes	7.25(1.22)	0.018	1.99 (1.99)	0.007	1.59(1.65)	
No	6.97(1.55)		1.57 (1.82)		1.38(1.67)	0.119

### Knowledge on oral health with DMFT score and Decayed score

Total decayed score was higher among those who did not have knowledge that fluoride could prevent decay compared to those who had knowledge about it. (Knowledge about fluoride prevents decay: mean = 1.21 (SD = 1.54) versus No knowledge: mean = 2.13 (SD = 2.13); p = 0.029). Those who did not have knowledge that gum diseases would affect teeth had higher decayed score compared to those who had knowledge. (Knowledge about gum disease affects teeth mean = 1.37 (SD = 1.59) versus No knowledge: mean = 2.30 (SD = 2.02); p = 0.015). (Table 3).

**Table 3:** Knowledge on oral health affecting DMFT score and Decayed score (P-value by ANOVA)

Variables	Total DMFT score Mean (SD)	p value	Total decayed score Mean (SD)	p value
*Regular brushing preventsdecay*				
Yes	1.69 (1.89)	0.187	1.44 (1.67)	0.178
No	1.22(1.35)		0.94(1.16)	
Don’t know	2.10 (1.99)		1.77(1.71)	
*Fizzy drinks affect teeth*				
Yes	1.67(1.79)	0.452	1.41(1.58)	0.439
No	1.96(2.23)		1.68(1.84)	
Don’t know	1.68(1.97)		1.44(1.81)	
*Fluoride prevents decay*				
Yes	1.53(1.94)	0.142	1.21(1.54)	0.029
No	2.33(2.20)		2.13(2.13)	
Don’t know	1.73(1.85)		1.48(1.67)	
*Gum disease causes gum bleed*				
Yes	1.71(1.92)	0.960	1.45(1.67)	0.996
No	1.61(1.64)		1.43(1.53)	
Don’t know	1.69(1.79)		1.44(1.69)	
*Gum disease affect teeth*				
Yes	1.65(1.85)	0.055	1.37(1.59)	0.015
No	2.60(2.16)		2.30(2.02)	
Don’t know	1.88(2.01)		1.70(1.94)	
*Proper brushing prevent decay*				
Yes	1.70(1.88)	0.823	1.45(1.67)	0.976
No	2.14(1.57)		1.57(1.51)	
Don’t know	1.73(2.10)		1.42(1.75)	
*Sugar destroys teeth*				
Yes	1.70(1.87)	0.953	1.44(1.64)	0.964
No	1.83(2.55)		1.43(2.25)	
Don’t know	1.70(1.82)		1.51(1.69)	
*Decay destroys teeth*				
Yes	1.70(1.88)	0.801	1.45(1.66)	0.964
No	1.29(2.98)		1.29(2.98)	
Don’t know	1.81(1.80)		1.47(1.46)	
*Healthy teeth mean strong and caries free*				
Yes	1.68(1.88)	0.487	1.44(1.68)	0.960
No	1.86(1.51)		1.57(1.50)	
Don’t know	2.02(2.09)		1.45(1.63)	

### Practice of oral health with DMFT score, decayed score and total knowledge score

The decayed score as tooth decaying in this study was 59.7%. Those who brushed their teeth two times a day had higher knowledge scores compared to those who brushed sometimes, once daily, two times a day and more than two times a day. (Brushing twice a day mean = 7.22 (SD = 1.34) versus more sometimes: mean = 6.76 (SD = 2.00). Those who used fluoridated toothpaste had higher knowledge scores compared to those who did not use fluoridated toothpaste (Fluoridated toothpaste: mean = 7.58 (SD = 1.32) versus non fluoridated toothpaste: mean = 7.03 (SD = 1.62); p < 0.001).

School children who had the practice of regular mouth gargling had higher knowledge scores compared to those who never had such practice. (Mouth gargled always = 7.13 (SD = 1.39) versus Never: mean = 6.27 (SD = 2.07); p = 0.002). The school children who cleaned their tongue always had higher knowledge scores compared to those who never cleaned their tongue (Tongue cleaned always: mean = 7.19 (SD = 1.40) versus never cleaned tongue: mean = 6.57 (SD = 1.53; p = 0.009). (Table 4).

**Table 4:** Practice on oral health on total knowledge score, DMFT score and Decayed score

Characteristics	Total knowledge score Mean (SD)	p value	Total DMFT score Mean (SD)	p value	Decayed score Mean (SD)	p value
*Brushing frequency*						
Sometimes	6.76(2.00)	0.027*	1.68(1.81)		1.46(1.74*)*	0.694*
Once-daily	6.90(1.48)		1.58 (1.84)		1.36(1.65)	
Two times a day	7.22(1.34)		1.80 (1.95)	0.549*	1.52(1.70)	
More than 2 times a day	6.90(1.65)		1.75 (1.58)		1.30(1.34)	
*Brushing Time*						
Less than 1 min	7.17(1.38)	0.150*	1.86(2.16)		1.51(1.75)	0.582*
2–3 min	7.11(1.45)		1.63(1.77)	0.455*	1.38(1.60)	
More than 3 min	6.89(1.52)		1.72 (1.85)		1.52(1.71)	
*Brushing aid*						
Datiwan/other	6.50(0.57)	0.442**	2.75(3.77)	0.269**	2.75(3.77)	0.118**
Brush	7.06(1.46)		1.70(1.88)		1.44(1.65)	
*Brushing material*						
Powder/coal/others	6.46(1.56)	0.135**	1.69(1.88)	0.980**	1.08(1.65)	0.421**
Toothpaste	7.07(1.45)		1.71(1.89)		1.45(1.67)	
*Brush change*						
Every 1 month	7.04(1.54)		1.70(1.92)	0.770*	1.46(1.73)	0.797*
Every 3 month	7.04(1.42)	0.868*	1.72(1.88)		1.47(1.60)	
Every 6 month	7.21(1.32)		2.00(1.98)		1.59 (1.45)	
Bristle out of shape	7.13(1.23)		1.61(1.77)		1.31(1.61)	
*Paste type*						
Non fluoridated	7.03(1.62)	< 0.001*	1.73(2.19)	0.552*	1.71(1.51)	0.298*
Don’t know	6.83(1.44)		1.76(1.91)		1.51(1.72)	
Fluoridated	7.58(1.32)		1.58(1.79)		1.34(1.57)	
*Mouth gargle*						
Never	6.27(2.07)	0.002*	2.03(2.12)	0.254*	1.78(2.08)	0.445*
Sometimes	6.94(1.45)		1.97(1.93)		1.46(1.54)	
Always	7.13(1.39)		1.65(1.87)		1.42(1.65)	
*Tongue clean*						
Never	6.57(1.53)	0.009*	1.89(1.88)	0.471*	1.67(1.71)	0.215*
Sometimes	6.99(1.49)		1.65(1.80)		1.45(1.71)	
Always	7.19(1.40)		1.67(1.92)		1.37(1.64)	

* One-way ANOVA

** t-test

### Association of socio-economic characteristics, knowledge and practices with total DMFT scores

A multiple linear regression model was developed covering outcome variable as DMFT scores and independent variables as gender, education level of father, education level of mother, occupation of father, occupation of mother, previous exposure to oral health education, knowledge scores, availability of water, regular brushing habit, mouth gargle after meal, and tongue cleaning after meal. Females were more likely to have higher DMFT scores compared to males (β-coefficient = 0.43, 95% CI 0.13, 0.73, p = 0.005) and higher knowledge scores were negatively associated with DMFT scores (β-coefficient = − 0.09, 95% CI − 0.20, − 0.01, p = 0.047). (Table 5).

**Table 5:** Multiple Regression Analysis: Association of socio-economic characteristics, knowledge and practices with total DMFT

Characteristics	β-coefficient^1^	95% Confidence interval for β		P value
		Lower bound	Upper bound	
Gender (female)	0.43	0.13	0.73	0.005
Education level of father (below secondary)	0.08	− 0.08	0.24	0.302
Education level of mother (below secondary)	− 0.07	− 0.23	0.09	0.385
Occupation of father (Unemployed)	− 0.03	− 0.14	0.09	0.634
Occupation of mother (Unemployed)	0.06	− 0.03	0.16	0.206
Previous Exposure to OHE (no)	0.19	− 0.12	0.50	0.232
Total knowledge score	− 0.09	− 0.20	− 0.01	0.047
Availability of water (no)	0.02	− 0.70	0.74	0.962
Regular brushing habit (no)	0.12	− 0.15	0.39	0.37
Mouth gargle after meal (no)	− 0.22	− 0.51	0.08	0.147
Tongue cleaning after meal (no)	− 0.10	− 0.30	0.09	0.291

^1^Adjusted for of gender, education level of father, education level of mother, occupation of father, occupation of mother, previous exposure to oral health education, knowledge score, availability of water, regular brushing habit, mouth gargle after meal, and tongue cleaning after meal

## Discussion

Female school children were more likely to have higher DMFT scores in this study. This could be due to the reason that adolescent girls might have given low priority for health and education and the gender disparity remains highly prevalent in Nepali society. The prevalence is similar with the study conducted in public school children in Nepal, where prevalence of dental caries was 60% [16]. A report from WHO has shown that adolescent girl’s health needs were higher and they had limited information and resources of health promotion activities in Nepal [17]. Our result is similar with the study conducted in India, which showed the higher mean DMFT scores in female students compared with that of male students [18, 19]. Similar results were found in a study conducted in Qatar in 2014 [20]. However, our results are different from the study conducted on DMFT scores among adolescents in the United Kingdom, which has shown that there was no disparity among adolescents by gender in terms of their DMFT scores [21]. Moreover, a randomized controlled trial conducted in Nepal has also identified the need of intervention to improve oral health knowledge as the school children had poor oral health knowledge [22].

Higher knowledge scores on oral health influence on having lower DMFT scores in multi-variate analysis. Children’s knowledge scores on oral health were dependent on father’s education level. Parents might have given more attention to their children about oral health and sought more oral health services, thus improving their knowledge scores [23]. Our findings are supported by that studies, which have shown that parents’ education level was directly related to students’ higher knowledge scores on oral health [24, 25].

Furthermore, the descriptive analysis showed that the school children who visited health institutions for oral health services were found to have higher knowledge scores and higher DMFT scores. In addition, the total knowledge scores were higher among those who brushed properly, used fluoridated toothpaste, gargled their mouth and cleaned their tongue. Our results are similar with the study conducted among Iranian school children [26].

Knowledge about the need of using fluoridated toothpaste was negatively associated with DMFT scores among children. Children with lower knowledge scores on using fluoridated toothpaste might have limited use of fluoridated toothpaste leading to higher DMFT scores. Similar results were obtained in a study conducted in Mangalore City of India, which has shown the lower level of knowledge on fluoridated toothpaste [27]. In addition, a study conducted in a rural village in Nepal among Chepang children has also shown that a high number of children did not have knowledge on fluoridated toothpaste and its importance to prevent dental caries [14].

Children’s proper oral hygiene practices such as proper brushing, use of fluoridated toothpaste and gargling led to improved oral health knowledge scores. Oral health knowledge among children had influenced towards improved proper brushing habit and frequency in a study conducted in China [28]. In addition, this study also found that knowledge had a role in improving oral health practices such as gargling and cleaning the tongue. Another study showed that the children with a habit of proper teeth brushing frequency with fluoride containing toothpaste had a higher knowledge on oral health and lower DMFT score [29].

Our study also showed that students with higher knowledge score, who gargled their mouth and cleaned the tongue had lower DMFT score. This finding is supported by a study conducted in Ethiopia which has shown that better oral health practices led to lower DMFT scores [30].

Regarding the strength of this study, this study was the first study in Gandaki Province, Nepal, which examined DMFT index and oral health condition among school children. Despite this strength, we have some limitations as well. The factors studied in this study are based on the reported responses in the questionnaire filled by the school children. The response might have been affected with social desirability bias and school children might have reported positive aspects of knowledge and practices. To minimize this bias, the first author as a dentist himself and his research assistants facilitated the session before filling questionnaires and briefed the school children on all questions that were to be filled out. Further, the first author himself as a dental surgeon, conducted checkup for dental problems of school children to measure DMFT index. We selected the community schools purposively in the urban area and peri-urban area of Kaski district in Gandaki Province so findings may not be appropriate to generalize in other settings in Nepal. However, the socioeconomic status of the parents and the community school settings in Nepal are somehow similar to our study settings. Therefore, this study may be generalized in the country and other similar settings elsewhere.

In conclusion, this study found that female students and those with poorer knowledge on oral health had higher DMFT scores. For oral health promotion of school children, periodic dental check-up with education on brushing frequency, use of fluoridated paste, tongue cleansing and care of gum diseases are recommended.

## Data Availability

All data generated or analyzed during this study are included in this published article.
